# Prevalence of respiratory pathogens among hospitalised patients with acute respiratory infection during and after the COVID-19 pandemic in Shijiazhuang, China

**DOI:** 10.3389/fcimb.2024.1486953

**Published:** 2024-11-28

**Authors:** Pan-pan Zheng, Ya-nan Zhao, Zhi-kai Wang, Min-zhen Wang, Rong Li, Jing Zhang, Nan Li, Zi-feng Zhang, Rui-juan Rong, Yi-chan Sun, Zan-chao Liu

**Affiliations:** ^1^ Hebei Key Laboratory of Basic Medicine for Diabetes, Shijiazhuang Second Hospital, Shijiazhuang, Hebei, China; ^2^ Shijiazhuang Technology Innovation Center of Precision Medicine for Diabetes, Shijiazhuang Second Hospital, Shijiazhuang, Hebei, China; ^3^ School of Clinical Medicine, Hebei Medical University, Shijiazhuang, Hebei, China; ^4^ Institute of Epidemiology and Health Statistics, School of Public Health, Lanzhou University, Lanzhou, Gansu, China

**Keywords:** COVID-19, respiratory pathogens, non-classical microorganisms, ARTIs/ILI, epidemiology

## Abstract

**Background:**

The COVID-19 pandemic and the resulting non-pharmaceutical interventions (NPIs) have led to changes in the epidemiology of other respiratory pathogens. This study was conducted to explore the epidemiological characteristics of 13 respiratory pathogens, including 11 respiratory viruses and 2 non-classical microorganisms, in hospitalised patients with acute respiratory tract infections (ARTIs) and to compare the prevalence of respiratory pathogens during and after the COVID-19 pandemic.

**Methods:**

We conducted a single-centre retrospective study involving 8979 patients with ARTIs in Shijiazhuang City from December 2019 to December 2023. The GeXP analysis platform and multiple reverse transcription–PCR (mRT–PCR) technology were used to simultaneously detect 13 respiratory pathogens. The ARIMA model was constructed to predict the pathogen detection rate in each quarter of Shijiazhuang City in the next 2 y.

**Results:**

Among the 8979 patients, 4169 (46.43%) tested positive for respiratory pathogens. The total pathogen detection rate rebounded in the year after the COVID-19 pandemic. After the COVID-19 pandemic, the positive rates in men were slightly higher than those in women and the positive rates in spring and winter were significantly higher than those in summer. The dominant pathogens during the COVID-19 pandemic were Influenza A viru (InfA; 24.08%) and Human Rhinovirus (HRV; 21.77%), and after the COVID-19 pandemic were InfA (27.92%) and H3 (21.17%). During the COVID-19 pandemic, InfA and HRV frequently occurred in all age groups. After the COVID-19 pandemic, InfA and Seasonal Influenza virus H3N2 (H3) frequently occurred in all age groups.

**Conclusions:**

A series of NPIs introduced by the Chinese government during the COVID-19 pandemic had a significant impact on acute upper respiratory pathogenic infections. After the withdrawal of the NPIs, the spectrum of respiratory pathogens changed.

Nearly 30% of hospitalised cases and 30%–60% of outpatient visits are related to respiratory infections (Albogami et al., 2018). Acute respiratory tract infections (ARTIs) are responsible for the high childhood morbidity and mortality in developing countries and are a major global health burden (World Health Organization, 2019). Viral ARTIs are one of the leading causes of hospital and outpatient visits in children and the elderly (Huang et al., 2018). Respiratory virus infection is one of the main causes of ARTIs, particularly upper respiratory tract infections, where viral infection accounts for 70%–80% of cases (Woodhead et al., 2011). An epidemiological investigation of respiratory pathogens is conducive to recovery from infection and control of pathogen outbreaks to avoid wider spread (Giamberardin et al., 2016; Ojuawo et al., 2020).

Because of the non-specific symptoms and the lack of rapid and sensitive diagnostic methods, including antigen-based testing and viral isolation culture, the use of empirical antibiotics in patients with viral ARTIs not only delays the time for treatment but also increases resistance (Benezit et al., 2020). The recommended method for diagnosing respiratory virus infections is nucleic acid testing, i.e. molecular detection methods, the use of which will reduce the turnaround time of diagnosis and will enable the detection of multiple pathogens simultaneously (Bibby et al., 2022). We used the GeXP genetic analysis platform and multiple reverse transcription–PCR (mRT–PCR) method (Wang et al., 2021) to simultaneously detect 13 major respiratory viruses and non-classical microorganisms.

The epidemiological pattern of respiratory pathogens is easily affected by factors such as the environment, geographical region, climate, human mobility and socioeconomic status (Albogami et al., 2018; Wu et al., 2023). In December 2019, the novel coronavirus (SARS-CoV-2) was first detected in China. Because humans have no natural immunity to the virus, COVID-19 eventually became a pandemic even with strict lockdown measures (Tan et al., 2022). SARS-CoV-2 infection can be transmitted by aerosols and respiratory droplets, sharing the same route of transmission as that of other respiratory pathogens (Zeng et al., 2021; Al et al., 2023). During the COVID-19 pandemic, the Chinese government used effective measures to treat COVID-19 patients and implemented non-pharmaceutical interventions (NPIs), including social distancing, closing schools, wearing masks, restricting travel, strengthening personal hygiene and closing borders, to curb disease transmission (Wu et al., 2023) while reducing the spread of viruses such as influenza A and B (Al et al., 2023). After the withdrawal of the NPIs in 2022–2023, viral ARTI-related hospitalisations increased and the influenza virus is currently co-circulating with SARS-CoV-2 in the UK (Nguyen et al., 2023), however, the situation in the Shijiazhuang area of China is unclear. This study analysed the incidence rate, gender, age of onset, seasonal changes of respiratory pathogens, and changes during and after the COVID-19 outbreak in all age groups.

## Methods

### Study population

We enrolled 8979 patients with suspected ARTIs who were hospitalised in Shijiazhuang Second Hospital from December 2019 to December 2023. The inclusion criteria were the following: (1) disease duration less than 3 days accompanied by cough or sore throat, nasal congestion, runny nose, sputum and other upper respiratory tract symptoms; (2) complete data. The exclusion criteria were the following: (1) acute respiratory inflammation caused by non-infectious factors; (2) parenchymatous organs or haematopoietic stem cell transplantation, immunodeficiency diseases such as human immunodeficiency virus infection, and cancer chemotherapy; and (3) incomplete data or patients not agreeing to participate in this study. This study was approved by the Ethics Committee of Shijiazhuang Second Hospital, and oral informed consent was obtained from all patients.

### Clinical data and sample collection

Demographic data were collected from the laboratory information system, including sex, age, clinical diagnosis, date of hospitalisation and date of pathogen testing. Nasopharyngeal swab samples were collected by professional clinicians and submitted for examination within 2 h. The collection of clinical data was de-identified and anonymous.

### GeXP-based multiple reverse transcription–polymerase chain reaction

A nucleic acid extraction BD-Micro kit (ZD Biotech, Zhejiang, China) was used to extract nucleic acid according to the manufacturer’s instructions. Multiple detection kits for 13 respiratory pathogens (ZD Biotech, Zhejiang, China) were used for respiratory pathogen detection. The multiple reverse transcription–PCR (mRT–PCR) reaction system and procedure are described in the kit instructions. The mRT–PCR products were separated in the GenomeLab™ GeXP (Beckman Coulter, USA) platform according to fragment size and migration rate and were analysed using the fluorescence signal intensity data. The 13 pathogens included Influenza A viru (InfA), Influenza A virus H1N1 (InfAH1N1), Seasonal Influenza virus H3N2 (H3), Influenza B virus (InfB), Human Adenovirus (HADV), Boca virus (Boca), Human Rhinovirus (HRV), Human Parainfluenza virus (HPIV), Human Coronavirus (HCOV), Human Respiratory Syncytial virus (HRSV), Human Metapneumovirus (HMPV), Mycoplasma Pneumoniae (MP) and Chlamydia (Ch). Quality control was performed during pathogen testing.

### Statistical analysis

The SPSS 21.0 statistical software and GraphPad Prism 5 were used to process and analyses the data. Categorical variables were compared using the chi-square test or Fisher’s exact test. A bilateral *P* value of <0.05 was considered statistically significant. The base, tseries, forecast and other software packages of R4.0.3 were used to construct the ARIMA prediction model for time series analysis, and the smaller the MAPE value was, the better was the fitting effect. Positive rate or positive detection rate refers to the frequency of positive detections. Positive proportion refers to the positive detection of a pathogen in the positive detection of all 13 pathogens. Positive cases refers to the number of positive patients.

## Results

### Characteristics of the respiratory pathogenic infection

A total of 8979 cases were collected from inpatients with ARTIsadmitted to the hospital, of whom 4801 (53.47%) were males and 4178 (46.53%) were females. The patient age range was from 9 months to 105 y. The socio-demographic variables are summarised in **Table 1**. A total of 4169 cases tested positive for respiratory pathogens, and the total positive detection rate was 46.43% (4169/8979). The positive detection rate of the InfAH1N1 virus and total infection rate in males were significantly higher than those in females (1.7% vs. 1.0%, *P* = 0.004; 24.15% vs. 22.29%, *P* = 0.020), and the positive detection rate of the H3 virus in females was significantly higher than that in males (9.3% vs. 7.9%, *P* = 0.042). The total pathogen detection rate was highest in the age group of 0–4 y (102.1%). With the exception of InfAH1N1, HCOV and Ch, there were significant differences in pathogen detection rates among different age groups (*P* < 0.001).

**Table 1:** Characteristics of respiratory pathogen detection in hospitalized patients of different gender and age groups.

	Gender			Age n (%)							
	Male n (%) n=4801	Female n (%) n=4178	*P*	0-4y n (%) n=756 (M:F=1.2)	5-18y n (%) n=1183 (M:F=)1.2	19-49y n (%) n=1250 (M:F=1.1)	50-64y n (%) n=1760 (M:F=1.0)	65-74y n (%) n=1836 (M:F=1.3)	75-84y n (%) n=1320 (M:F=1.2)	≥85y n (%) n=874 (M:F=1.2)	*P*
InfA	591 (12.3)	545 (13.0)	0.360	143 (18.9)	205 (17.3)	165 (13.2)	207 (11.8)	221 (12.0)	122 (9.2)	73 (8.3)	<0.001
InfAH1N1	84 (1.7)	42 (1.0)	0.004	2 (0.3)	17 (1.4)	19 (1.5)	38 (2.2)	27 (1.5)	15 (1.1)	8 (0.9)	0.802
H3	381 (7.9)	387 (9.3)	0.042	94 (12.4)	144 (12.2)	111 (8.9)	127 (7.2)	149 (8.1)	90 (6.8)	53 (6.1)	<0.001
InfB	62 (1.3)	74 (1.8)	0.069	10 (1.3)	24 (2.0)	56 (4.5)	18 (1.0)	14 (0.8)	5 (0.4)	9 (1.0)	<0.001
HADV	121 (2.5)	98 (2.4)	0.632	43 (5.7)	81 (6.8)	20 (1.6)	22 (1.2)	27 (1.5)	18 (1.4)	8 (0.9)	<0.001
Boca	28 (0.6)	20 (0.5)	0.563	31 (4.1)	2 (0.2)	3 (0.2)	3 (0.2)	5 (0.3)	3 (0.2)	1 (0.1)	<0.001
HRV	290 (6.0)	228 (5.5)	0.277	102 (13.5)	131 (11.1)	63 (5.0)	75 (4.3)	57 (3.1)	53 (4.0)	37 (4.2)	<0.001
HPIV	83 (1.7)	82 (2.0)	0.423	70 (9.3)	32 (2.7)	4 (0.3)	18 (1.0)	20 (1.1)	10 (0.8)	11 (1.3)	<0.001
HCOV	57 (1.2)	51 (1.2)	0.923	14 (1.8)	16 (1.4)	12 (1.0)	11 (0.6)	19 (1.0)	23 (1.7)	13 (1.5)	0.853
HRSV	125 (2.6)	118 (2.8)	0.558	144 (19.0)	40 (3.4)	5 (0.4)	9 (0.5)	15 (0.8)	17 (1.3)	13 (1.5)	<0.001
HMPV	120 (2.5)	132 (3.2)	0.073	84 (11.1)	72 (3.6)	16 (1.3)	31 (1.8)	26 (1.4)	15 (1.1)	8 (0.9)	<0.001
MP	221 (4.6)	220 (5.3)	0.171	33 (4.4)	316 (26.7)	52 (4.2)	24 (1.4)	14 (0.8)	2 (0.1)	0 (0.0)	<0.001
Ch	5 (0.1)	4 (0.1)	1.000	2 (0.3)	1 (0.1)	3 (0.2)	0 (0.0)	3 (0.2)	0 (0.0)	0 (0.0)	0.080
Total	2168 (45.2)	2001 (47.9)	0.119	772 (102.1)	1081 (91.4)	529 (42.3)	583 (33.1)	597 (32.5)	373 (28.3)	234 (26.8)	<0.001

### Distribution of the pathogen positive detection rate in different years

**Figure 1:**
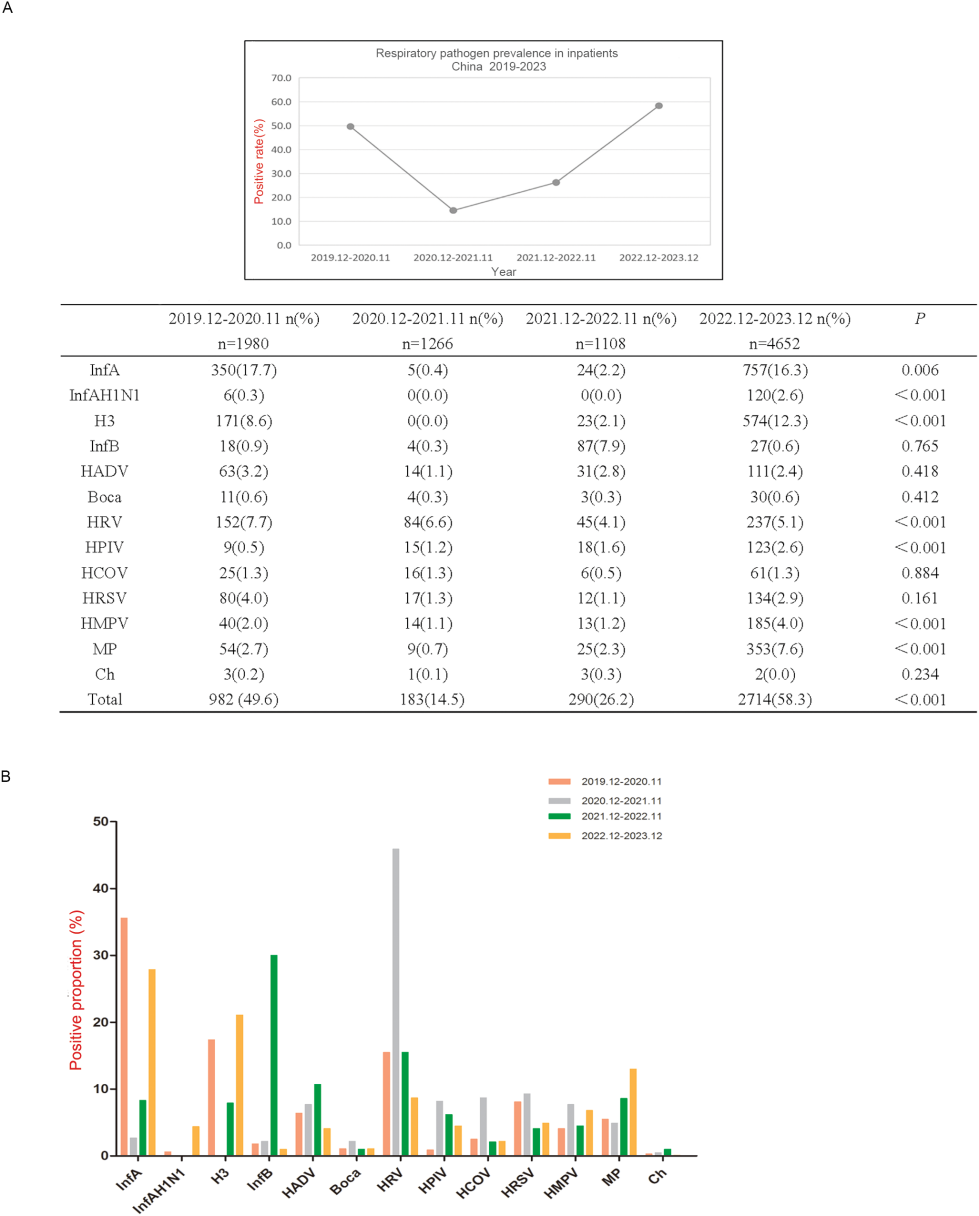
Respiratory pathogen detection among hospitalized patients in 2019.12-2023.12. **(A)** Respiratory pathogen detection rate from December 2019 to December 2023. **(B)** Differences of positive rates of each respiratory pathogen in four time periods from December 2019 to December 2023. Positive rate refers to the frequency of positive detections in the current year. Positive proportion refers to the positive detection of a pathogen in the positive detection of all 13 pathogens.

In December 2019, the first pandemic strain 19A was found in Wuhan, China, and since then, China’s epidemic prevention and control has started. China launched an emergency management mechanism for the first time and began to implement strict epidemic prevention and control measures until December 2022,when the State Council’s epidemic prevention measures to optimise the ‘new 10’ (Rongfeng et al., 2024). Therefore, we collected data for 3 y during the COVID-19 outbreak and for 1 y after the COVID-19 pandemic, as shown in **Figure 1**.

The number of pathogens detected in the first 3 y ranged from 1000 to 2000 and rebounded to more than 4,600. The positive detection rates of InfA, InfAH1N1, H3, HRV, HPIV, HMPV and MP were significantly different among the four time periods, which were all higher from December 2022 to December 2023 than from the other three time periods, except for HRV (*P* < 0.01) (**Figure 1A**). From December 2019 to November 2020, the main pathogenic infections were InfA, H3 and HRV; from December 2020 to November 2021, the pathogenic infection was mainly HRV; from December 2021 to November 2022, the main pathogenic infections were InfB and HRV; and from December 2022 to December 2023, the pathogenic infections were mainly InfA, H3 and MP (**Figure 1B**).

### Monthly activity patterns of the respiratory pathogen positive rate

**Figure 2:**
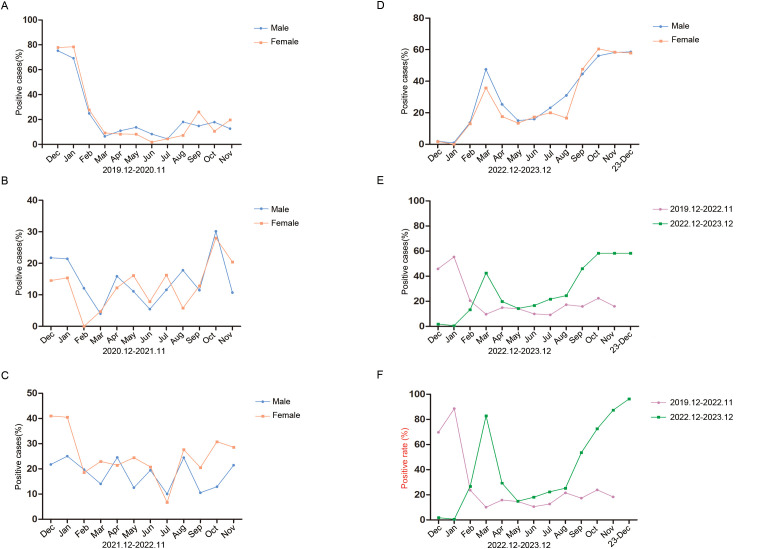
Monthly activity patterns of respiratory pathogens positive rate. **(A)** Monthly activity patterns of respiratory pathogens positive rate from December 2019 to November 2020. **(B)** Monthly activity patterns of respiratory pathogens positive rate from December 2020 to November 2021. **(C)** Monthly activity patterns of respiratory pathogens positive rate from December 2021 to November 2022. **(D)** Monthly activity patterns of respiratory pathogens positive rate from December 2022 to December 2023. Different monthly activity patterns of respiratory pathogens positivity rates **(E)** and positive detection rates **(F)** during the two time periods of December 2019 to November 2022 and December 2022 to December 2023. Positive cases (%) refers to the number of positive patients in the total number of patients tested. Positive rate refers to the frequency of postive detections.

We analysed the monthly distribution of respiratory pathogens by gender and during and after the COVID-19 outbreak in the study period, as shown in **Figure 2**. In the four time periods, there was no significant difference in the positive rates of males and females. In the first year of the pandemic, the positive rate of males was highest in December (75.31%) and lowest in July (4.44%), and the positive rate of females was highest in January (78.43%) and lowest in June (1.79%) (**Figure 2A**). In the second year, the positive rate of males was highest in October (30.16%) and lowest in March (4.00%), and the positive rate of females was highest in October (28.00%) and lowest in February (0.00%). The highest positivity rates for males and females were much lower than those in the first year (**Figure 2B**). In the third year, the positive rate of males was highest in January (25.00%) and lowest in July (10.00%), and the positive rate of females was highest in December (40.98%) and lowest in July (6.67%) (**Figure 2C**). In the fourth year, the positive rates were higher in spring and winter than in summer and autumn. The positive rate of males was highest in December (58.54%) and lowest in January (0.93%). The positive rate of females was highest in October (60.53%) and lowest in January (0.00%). The total positive rate of males was significantly higher than that of females (*P* < 0.001) (**Figure 2D**).

We then statistically analysed the changes in pathogen positive rates (only one count of overlapping infections) and positive detection rates (including overlapping infections) during and after the COVID-19 pandemic. From December 2019 to November 2022, the positive rate of pathogens showed a downwards trend without seasonal dependence, and from December 2022 to December 2023, the positive rate of pathogens showed an upwards trend and the positive rates in spring and winter were higher than those in summer and autumn (**Figure 2E**). The positive detection rate of pathogens showed the same trend (**Figure 2F**). These results suggest that an increase in pathogen positive rates is accompanied by an increase in overlapping infections.

### Monthly distribution of 13 respiratory pathogens

**Figure 3:**
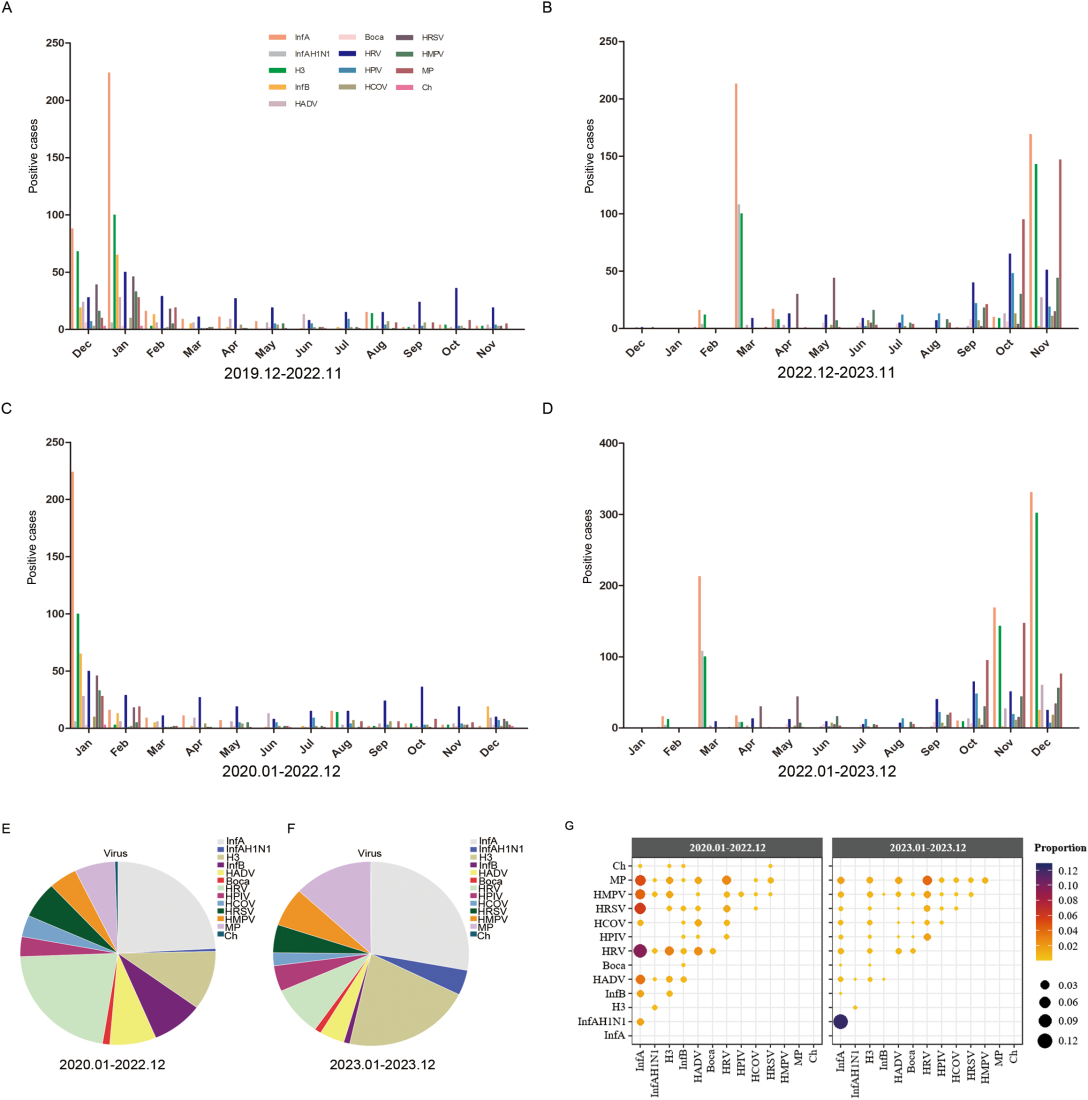
The monthly distribution of different respiratory pathogens. **(A)** The monthly distribution of different respiratory pathogens from December 2019 to November 2022. **(B)** The monthly distribution of different respiratory pathogens from December 2022 to November 2023. **(C)** The monthly distribution of different respiratory pathogens from January 2022 to December 2022. **(D)** The monthly distribution of different respiratory pathogens from January 2023 to December 2023. **(E)** The proportions of different pathogens from January 2022 to December 2022. **(F)** The porportions of different pathogens from January 2023 to December 2023. **(G)** The proportion of each co-infection pair among all co-infected samples shown by color and size from January 2022 to December 2022 and January 2023 to December 2023. Positive cases refers to the number of positive patients.

The monthly distribution of each respiratory pathogen during and after the COVID-19 pandemic is shown in **Figure 3**. During the COVID-19 pandemic, the peak of InfA and H3 virus infection occurred in winter (January and December) and the lowest activity was in spring and summer (from March to July). There was no clear seasonal distribution for HRV, and infections were high throughout the year (**Figure 3A**). However, as shown in **Figure 3C**, it can be seen that the higher number of infections in December was mainly contributed by December 2019. After the COVID-19 pandemic, InfA and H3 virus infections peaked in spring (March) and winter (October), with the lowest activity in summer and autumn (from May to September). MP dramatically increased in winter (from October to November). HRV infection increased significantly in autumn and winter (from September to November). HRSV had a small increase in April and May. The number of positive samples for the other pathogens was smaller but significantly higher than that during the COVID-19 pandemic, and the peak of infection was also in autumn and winter (**Figure 3B**). However, as shown in **Figure 3D**, it can be seen that because of the seasonal advantage of winter, the number of pathogenic infections in December 2023 was still increasing dramatically. Since the first positive case of COVID-19 was found in Shijiazhuang (also Hebei Province) in January 2020, and nucleic acid inspection was lifted from January 2023, and according to the actual monthly change distribution of respiratory viruses, we strictly referred to the 2020.01-2022.12 as 3 y of pandemic, and the 2023.01-2023.12 as 1 y after the pandemic.

During the COVID-19 pandemic, the total number of positive pathogens was 1217 and the dominant pathogens were InfA and HRV, accounting for 24.08% and 21.77% of the total number of positive pathogens, respectively, followed by H3, accounting for 10.35% (**Figure 3E**). After COVID-19 pandemic, the total number of positive pathogens was 2711 and the dominant pathogens were InfA and H3, accounting for 27.92% and 21.17% of the total number of positive pathogens, respectively, followed by MP, accounting for 13.02% (**Figure 3F**). Both during and after the COVID-19 pandemic, InfA/H3 had the highest proportion of co-infection among all tested samples, respectively, accounting for 35.13% (124/353) and 58.81% (574/976), which was not shown in the figure because it was much higher than the other co-infections (**Figure 3G**).

### Comparison of pathogenic epidemiological characteristics during and after the COVID-19 pandemic

**Figure 4:**
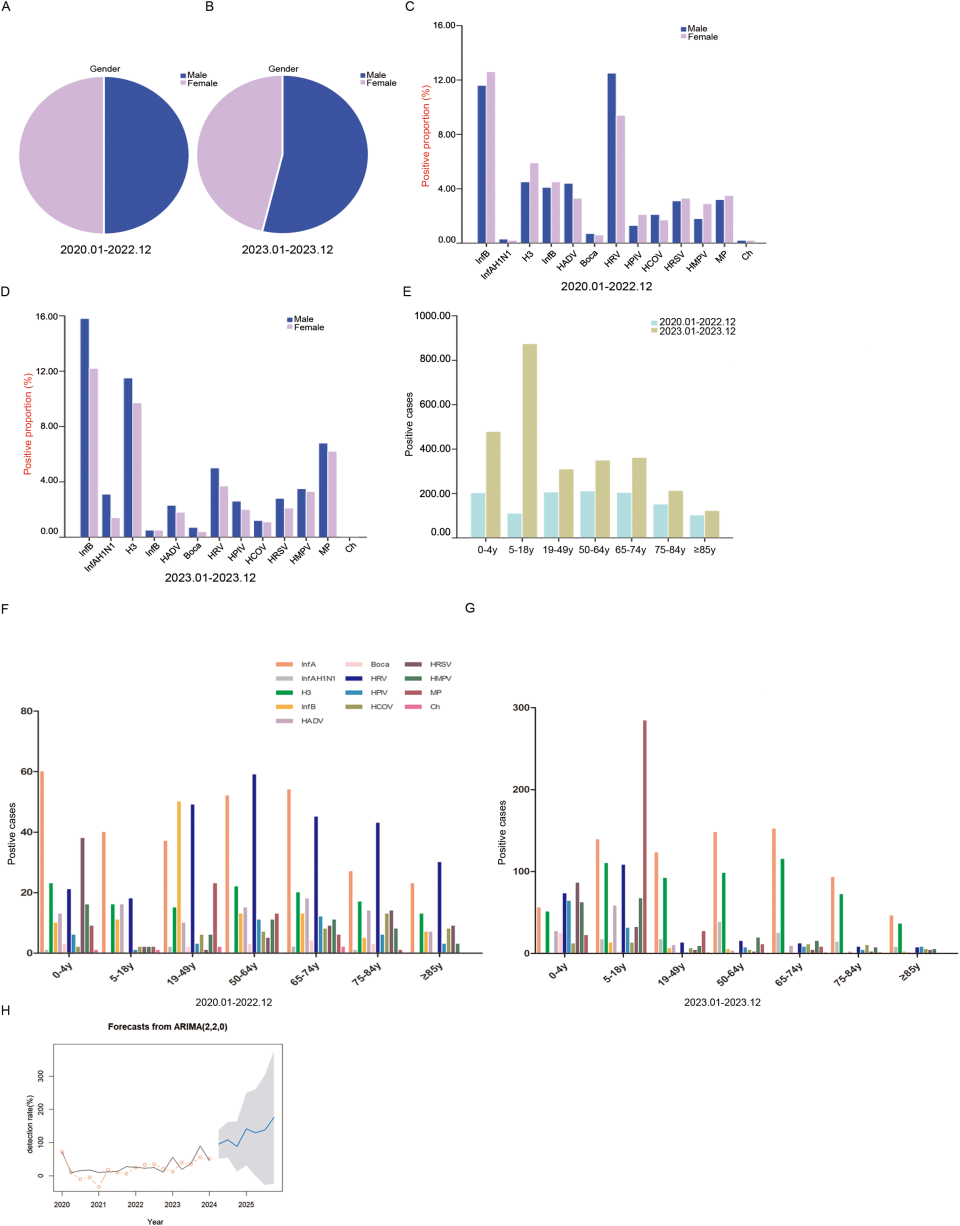
The demographic characteristics of respiratory pathogens during and after the COVID-19. **(A)** The proportions of different sexes among positive samples during the COVID-19. **(B)** The proportions of different sexes among positive samples after the COVID-19. **(C)** The proportions of different pathogens in sex-specific positive samples during the COVID-19. **(D)** The proportions of different pathogens in sex-specific positive samples after the COVID-19. **(E)** The proportions of different ages of positive samples. **(F)** The distribution of pathogens in positive samples among different ages during the COVID-19. **(G)** The distribution of pathogens in positive samples among different ages after the COVID-19. **(H)** The AMIRA time series model predicted the respiratory pathogen detection rates from April 2024 to December 2025. The black line is the actual value, the red circle is the fitting value, the blue is the predicted value, and the gray area is the 95% confidence interval. Positive proportion refers to the positive detection of a pathogen in the positive detection of all 13 pathogens. Positive cases refers to the number of positive patients.

During the COVID-19 pandemic, male and female positive samples accounted for 49.83% and 50.17%, respectively, and the difference was not statistically significant (*P* = 0.861) (**Figure 4A**). After the COVID-19 outbreak, the proportion of males in positive samples was significantly higher than that of females (53.78% vs. 46.22%, *P* = 0.001) (**Figure 4B**). During the COVID-19 pandemic, HRV and InfA were the main pathogens in males. InfA and HRV were the main pathogens in females (**Figure 4C**). After the COVID-19 pandemic, the main pathogens in males and females were InfA, H3 and MP. The proportions of InfA, H3 and MP in males were higher than those in females (**Figure 4D**). The number of positive samples in all age groups was higher after the COVID-19 outbreak than during the pandemic (**Figure 4E**). During the COVID-19 pandemic, InfA and HRV frequently occurred in all age groups. HRSV infection also frequently occurred in the age group of 0–4 y, and InfB and MP infections frequently occurred in the age group of 19–49 y and were much higher than those in the other age groups (**Figure 4F**). After the COVID-19 pandemic, InfA and H3 frequently occurred in all age groups. HRSV infection also frequently occurred in the age group of 0–4 y, and MP infection frequently occurred in the age group of 5–18 y and was much higher than that in the other age groups (**Figure 4G**). ARIMA time series prediction modelling was performed on the overall respiratory pathogenic infections, and the total predicted pathogen detection rates for seven quarters from April 2024 to December 2025 in Shijiazhuang City were obtained. The optimal model of acute upper respiratory tract infection in Shijiazhuang was ARIMA (2,2,0), and MAPE was 75.92 (**Figure 4H**).

## Discussion

Respiratory pathogens are common in the general population, placing a heavy economic burden on the public health system. As a double-edged sword, the national action plan during the COVID-19 period while controlling the spread of the pandemic can easily lead to the continuous recurrence of respiratory pathogens due to the lack of long-term immunity, and it is urgent to adjust the immunisation strategy in time. This study focused on the epidemiological characteristics of respiratory pathogens causing ARTIs in North China during and after the COVID-19 pandemic period from December 2019 to December 2023 and compared the changes in the pathogen epidemic spectra before and after the COVID-19 pandemic to help actively cope with the ‘immunity debt’ after the COVID-19 pandemic.

Most pre-COVID-19 studies showed that men were more susceptible than women, possibly because of more social and hormonal influences (Richter et al., 2016; Wang et al., 2016; Albogami et al., 2018). We found that the positive detection rate of InfAH1N1 and total infection rate in males were significantly higher than those in females and the positive detection rate of the H3 virus in females was significantly higher than that in males,which was also different from the results reported during the COVID-19 period that boys may be more susceptible to respiratory pathogenic infection than girls (Yassine et al., 2020), which may be related to the impact of the NPIs on the trajectory of human flow. We found that the total pathogen detection rates were highest in the age group of 0–4 y, which was consistent with the facts that despite general population susceptibility, children are the main source of respiratory infections and that younger children are more susceptible to viral infections than older children (Williams et al., 2002; Wan et al., 2023). The detection rates of most pathogens were different between age groups, so the infection appeared to be closely related to age. We found that compared with the downwards trend during the COVID-19 pandemic, the total pathogen detection rate rebounded after the COVID-19 outbreak, similar to what was reported in a study in Guangzhou, China (Zeng et al., 2021). Our results are consistent with recent literature reporting that HRVs are very prevalent viruses during the COVID-19 pandemic and the rebound of influenza viruses after COVID-19 pandemic (Lai et al., 2022; Regina et al., 2024). ARTIs are the leading cause of medical visits in winter and spring for all age groups before COVID-19 (Li et al., 2019). Our results showed that there was no significant seasonal distribution of positive rates of patients regardless of gender during the COVID-19 pandemic, with a dramatic decrease beginning in January 2020. However, after the COVID-19 pandemic, the infection rate of men were slightly higher than that of women, and the infection rate of spring and winter were significantly higher than that of summer. The first peak of respiratory infections after the COVID-19 outbreak was in March. These results were consistent with many studies showing large reductions in outpatient and inpatient visits in many countries in 2020 (the COVID-19 began) (Friedrich et al., 2021; Pines et al., 2021). This suggests that respiratory pathogens begin to resume their original transmission characteristics after the NPIs were withdrawn.

It has been reported that the NPIs during the COVID-19 pandemic typically slow down and alleviate the spread of respiratory pathogens in populations (Kaleta et al., 2022). A series of comprehensive new public health preventive measures in response to the COVID-19 pandemic have resulted in significant reductions in the prevalence of non-SARS-CoV-2 infections (Olsen et al., 2021; Ren et al., 2023). Rare studies of pathogenic infection characteristics after the COVID-19 pandemic have been reported. Our study showed that the dominant pathogens during the COVID-19 pandemic were InfA (24.08%) and HRV (21.77%) and the dominant pathogens after COVID-19 were InfA (27.92%) and H3 (21.17%), followed by MP (13.02%). InfA/H3 had the highest proportion of co-infections among all tested samples during (35.13%) and after (58.81%) the COVID-19 pandemic. These results demonstrated that NPIs can indeed be very effective against influenza (Wan et al., 2023; Gao et al., 2024), influenza virus infection resumed after the withdrawal of the NPIs and the transmission of MP in the population is significantly enhanced. During the COVID-19 pandemic, there was no significant difference in the proportion of positive samples between males and females and the infection rate of men were slightly higher than that of women after the COVID-19, indicating that NPIs changed the distribution of sexual respiratory pathogens and the distribution of sexual respiratory pathogens recovered after the withdrawal of the NPIs. Our results showed that during the COVID-19 pandemic, InfA and HRV frequently occurred in all age groups. HRSV infection also frequently occurred in the age group of 0–4 y, and InfB and MP infections frequently occurred in the age group of 19–49 y and were much higher than those in the other age groups. After the COVID-19 pandemic, InfA and H3 frequently occurred in all age groups. HRSV infection also frequently occurred in the age group of 0–4 y, and MP infection frequently occurred in the age group of 5–18 y and was much higher than that in the other groups. Previous epidemiological survey data show that HRSV is the most common respiratory virus in children in Vietnam (Lu et al., 2020), and another study in Beijing found that influenza A is the most important virus, followed by HRSV (Li et al., 2020). However, our data showed that during the COVID-19 period, InfA was the most common virus in children in Shijiazhuang, followed by HRSV. After the COVID-19 pandemic, HRSV was the most important respiratory virus in children. In any case, HRSV was the primary pathogen in children aged 0–4 y during or after the COVID-19 pandemic, which is consistent with reports from China and other countries (Duan et al., 2021; Li et al., 2021; Wan et al., 2023). These results suggest that SARS-CoV-2 infection did not change the primary population of HRSV infection and that pathogenic infection varies by geographical characteristics or living environment as children’s playgrounds change from home or community to school after the outbreak. Another study in Sichuan, China, confirmed that SARS-CoV-2 had no effect on the positive rate of seven respiratory viruses, including HRSV, in children (Duan et al., 2021). Our results also revealed that young and middle-aged people should be alert to the prevalence of MP.

This study compared the prevalence of 13 pathogens during and after the COVID-19 pandemic and found that the removal of the NPIs after the pandemic resulted in the rebound of respiratory pathogens, which may cause ‘immunity debt’. At the same time, the time series model established using the data from December 2091 to January 2024 suggests that the positive detection rate of pathogens is still at a relatively high level in the short term, suggesting that clinical workers should strengthen the monitoring and management of non-SARS-CoV-2 infections. There are still some shortcomings, although our study applied the gold standard method of real-time PCR. First, there might be some viruses that were not taken into account. Second, our study was limited to the Shijiazhuang region and may not be applicable to other regions. Third, fewer outpatient visits during the COVID-19 pandemic might have resulted in lower test samples. Fourth, some factors that influence positive rates, including the influenza vaccine coverage, were not taken into account.

## Conclusion

This report is the first to identify the prevalence of respiratory pathogens in hospitalised patients of all ages during and after the COVID-19 pandemic in Shijiazhuang, which is generally consistent with other reports before, during and after the COVID-19 pandemic, demonstrating that non-drug interventions have a significant negative impact on the transmission of other respiratory pathogens. In the past 3 y of the COVID-19 outbreak, surveillance efforts mainly focused on SARS-CoV-2. Future studies need to combine SARS-CoV-2 with ARTIs to achieve comprehensive, active and sustained epidemiological surveillance and alert to ‘immunity debt’.

## Data Availability

The raw data supporting the conclusions of this article will be made available by the authors, without undue reservation.

## References

[B1] Al K. H. Meredith L. W. Al-Jardani A. Sajina F. Al S. I. Al H. R.. (2023). Time trend of respiratory viruses before and during the COVID-19 pandemic in severe acute respiratory virus infection in the Sultanate of Oman between 2017 and 2022. Influenza Other Respir. Viruses 17, e13233. doi: 10.1111/irv.13233 38098648 PMC10719608

[B2] Albogami S. S. Alotaibi M. R. Alsahli S. A. Masuadi E. Alshaalan M. (2018). Seasonal variations of respiratory viruses detected from children with respiratory tract infections in Riyadh, Saudi Arabia. J. Infect. Public Health 11, 183–186. doi: 10.1016/j.jiph.2017.06.001 28668655

[B3] Benezit F. Loubet P. Galtier F. Pronier C. Lenzi N. Lesieur Z.. (2020). Non-influenza respiratory viruses in adult patients admitted with influenza-like illness: a 3-year prospective multicenter study. Infection 48, 489–495. doi: 10.1007/s15010-019-01388-1 32056143 PMC7095392

[B4] Bibby H. L. de Koning L. Seiden-Long I. Zelyas N. Church D. L. (2022). A pragmatic randomized controlled trial of rapid on-site influenza and respiratory syncytial virus PCR testing in paediatric and adult populations. BMC Infect. Dis. 22, 854. doi: 10.1186/s12879-022-07796-3 36384484 PMC9667852

[B5] Duan Y. He J. Cui Y. Li W. Jiang Y. (2021). Characteristics and forecasting of respiratory viral epidemics among children in west China. Med. (Baltimore) 100, e25498. doi: 10.1097/MD.0000000000025498 PMC8078258 33879683

[B6] Friedrich F. Ongaratto R. Scotta M. C. Veras T. N. Stein R. T. Lumertz M. S.. (2021). Early impact of social distancing in response to coronavirus disease 2019 on hospitalizations for acute bronchiolitis in infants in Brazil. Clin. Infect. Dis. 72, 2071–2075. doi: 10.1093/cid/ciaa1458 32986818 PMC7543304

[B7] Gao Z. Wang Y. Yan L. Liu T. Peng L. (2024). Epidemiological characteristics of respiratory viruses in children during the COVID-19 epidemic in Chengdu, China. Microbiol. Spectr. 12, e261423. doi: 10.1128/spectrum.02614-23 PMC10783071 38051057

[B8] Giamberardin H. I. Homsani S. Bricks L. F. Pacheco A. P. Guedes M. Debur M. C.. (2016). Clinical and epidemiological features of respiratory virus infections in preschool children over two consecutive influenza seasons in southern Brazil. J. Med. Virol. 88, 1325–1333. doi: 10.1002/jmv.24477 26773605 PMC7167150

[B9] Huang H. S. Tsai C. L. Chang J. Hsu T. C. Lin S. Lee C. C. (2018). Multiplex PCR system for the rapid diagnosis of respiratory virus infection: systematic review and meta-analysis. Clin. Microbiol. Infect. 24, 1055–1063. doi: 10.1016/j.cmi.2017.11.018 29208560 PMC7128951

[B10] Kaleta M. Kesik-Brodacka M. Nowak K. Olszewski R. Sliwinski T. Zoltowska I. (2022). Long-term spatial and population-structured planning of non-pharmaceutical interventions to epidemic outbreaks. Comput. Oper Res. 146, 105919. doi: 10.1016/j.cor.2022.105919 35755160 PMC9212736

[B11] Lai S. Y. Liu Y. L. Jiang Y. M. Liu T. (2022). Precautions against COVID-19 reduce respiratory virus infections among children in Southwest China. Med. (Baltimore) 101, e30604. doi: 10.1097/MD.0000000000030604 PMC9477712 36123935

[B12] Li Y. Johnson E. K. Shi T. Campbell H. Chaves S. S. Commaille-Chapus C.. (2021). National burden estimates of hospitalisations for acute lower respiratory infections due to respiratory syncytial virus in young children in 2019 among 58 countries: a modelling study. Lancet Respir. Med. 9, 175–185. doi: 10.1016/S2213-2600(20)30322-2 32971018

[B13] Li Y. Reeves R. M. Wang X. Bassat Q. Brooks W. A. Cohen C.. (2019). Global patterns in monthly activity of influenza virus, respiratory syncytial virus, parainfluenza virus, and metapneumovirus: a systematic analysis. Lancet Glob Health 7, e1031–e1045. doi: 10.1016/S2214-109X(19)30264-5 31303294

[B14] Li Y. Wang J. Wang C. Yang Q. Xu Y. Xu J.. (2020). Characteristics of respiratory virus infection during the outbreak of 2019 novel coronavirus in Beijing. Int. J. Infect. Dis. 96, 266–269. doi: 10.1016/j.ijid.2020.05.008 32389850 PMC7204690

[B15] Lu L. Robertson G. Ashworth J. Pham H. A. Shi T. Ivens A.. (2020). Epidemiology and phylogenetic analysis of viral respiratory infections in Vietnam. Front. Microbiol. 11, 833. doi: 10.3389/fmicb.2020.00833 32499763 PMC7242649

[B16] Nguyen V. H. Ashraf M. Mould-Quevedo J. F. (2023). Estimating the impact of influenza vaccination of low-risk 50-64-year-olds on acute and ICU hospital bed usage in an influenza season under endemic COVID-19 in the UK. Hum. Vaccin Immunother. 19, 2187592. doi: 10.1080/21645515.2023.2187592 36912725 PMC10054290

[B17] Ojuawo O. B. Desalu O. O. Fawibe A. E. Ojuawo A. B. Aladesanmi A. O. Opeyemi C. M. (2020). Clinical and microbiological profile of adult inpatients with community acquired pneumonia in Ilorin, North Central, Nigeria. Afr Health Sci. 20, 1655–1668. doi: 10.4314/ahs.v20i4.18 34394226 PMC8351858

[B18] Olsen S. J. Winn A. K. Budd A. P. Prill M. M. Steel J. Midgley C. M.. (2021). Changes in influenza and other respiratory virus activity during the COVID-19 pandemic-United States, 2020-2021. Am. J. Transplant. 21, 3481–3486. doi: 10.1111/ajt.16049 34624182 PMC8653380

[B19] Pines J. M. Zocchi M. S. Black B. S. Carlson J. N. Celedon P. Moghtaderi A.. (2021). Characterizing pediatric emergency department visits during the COVID-19 pandemic. Am. J. Emerg. Med. 41, 201–204. doi: 10.1016/j.ajem.2020.11.037 33257144 PMC7682424

[B20] Regina M. I. C. Santos M. O. de Oliveira C. M. de Araujo K. M. de Souza G. Rezio G. S.. (2024). Rhinovirus infection and co-infection in children with severe acute respiratory infection during the COVID-19 pandemic period. Virulence 15, 2310873. doi: 10.1080/21505594.2024.2310873 38384141 PMC10885176

[B21] Ren L. Lin L. Zhang H. Wang Q. Cheng Y. Liu Q.. (2023). Epidemiological and clinical characteristics of respiratory syncytial virus and influenza infections in hospitalized children before and during the COVID-19 pandemic in Central China. Influenza Other Respir. Viruses 17, e13103. doi: 10.1111/irv.13103 36824393 PMC9895987

[B22] Richter J. Panayiotou C. Tryfonos C. Koptides D. Koliou M. Kalogirou N.. (2016). Aetiology of acute respiratory tract infections in hospitalised children in Cyprus. PLoS One 11, e147041. doi: 10.1371/journal.pone.0147041 PMC4720120 26761647

[B23] Rongfeng Z. Kai S. Fang X. Hongzhou L.. (2024). Changes in China's Prevention and control policies of the Novel Coronavirus Pneumonia Epidemic. Fudan J. (Medical Edition) 51, 109–114. Available online at: https://link.cnki.net/urlid/31.1885.r.20240108.1025.012

[B24] Tan M. P. Leong C. L. Pang Y. K. Razali R. M. Ismail A. I. Sam I. C.. (2022). Dearth of influenza among older adults admitted with respiratory symptoms in Malaysia during the coronavirus disease 2019 pandemic in 2021. Front. Med. (Lausanne) 9, 977614. doi: 10.3389/fmed.2022.977614 36300181 PMC9589354

[B25] Wan L. Li L. Zhang H. Liu C. Li R. Wu X.. (2023). The changing pattern of common respiratory viruses among children from 2018 to 2021 in Wuhan, China. Arch. Virol. 168, 291. doi: 10.1007/s00705-023-05891-7 37962775 PMC10645662

[B26] Wang H. Gu J. Li X. van der Gaast-de J. C. Wang W. He X.. (2021). Broad range detection of viral and bacterial pathogens in bronchoalveolar lavage fluid of children to identify the cause of lower respiratory tract infections. BMC Infect. Dis. 21, 152. doi: 10.1186/s12879-021-05834-0 33546631 PMC7864134

[B27] Wang H. Zheng Y. Deng J. Wang W. Liu P. Yang F.. (2016). Prevalence of respiratory viruses among children hospitalized from respiratory infections in Shenzhen, China. Virol. J. 13, 39. doi: 10.1186/s12985-016-0493-7 26952107 PMC4782311

[B28] Williams B. G. Gouws E. Boschi-Pinto C. Bryce J. Dye C. (2002). Estimates of world-wide distribution of child deaths from acute respiratory infections. Lancet Infect. Dis. 2, 25–32. doi: 10.1016/S1473-3099(01)00170-0 11892493

[B29] Woodhead M. Blasi F. Ewig S. Garau J. Huchon G. Ieven M.. (2011). Guidelines for the management of adult lower respiratory tract infections–full version. Clin. Microbiol. Infect. 17 Suppl 6, E1–E59. doi: 10.1111/j.1469-0691.2011.03672.x PMC7128977 21951385

[B30] World Health Organization (2019).International statistical classification of diseases and related health problems 10th revision. Available online at: https://icd.who.int/browse10/2019/en/.

[B31] Wu R. Zhang J. Mo L. (2023). Analysis of respiratory virus detection in hospitalised children with acute respiratory infection during the COVID-19 pandemic. Virol. J. 20, 253. doi: 10.1186/s12985-023-02218-5 37919789 PMC10623845

[B32] Yassine H. M. Sohail M. U. Younes N. Nasrallah G. K. (2020). Systematic review of the respiratory syncytial virus (RSV) prevalence, genotype distribution, and seasonality in children from the middle east and north Africa (MENA) region. Microorganisms 8 (5), 713. doi: 10.3390/microorganisms8050713 32403364 PMC7284433

[B33] Zeng Z. Guan W. Liu Y. Lin Z. Liang W. Liang J.. (2021). Different circulation pattern of multiple respiratory viruses in southern China during the COVID-19 pandemic. Front. Microbiol. 12, 801946. doi: 10.3389/fmicb.2021.801946 35154032 PMC8826816

