# Supplementary material for: Prevalence of respiratory pathogens among hospitalised patients with acute respiratory infection during and after the COVID-19 pandemic in Shijiazhuang, China
Source: Front Cell Infect Microbiol. 2024 Nov 28;14:1486953. doi: 10.3389/fcimb.2024.1486953 (PMC11635993; doi:10.3389/fcimb.2024.1486953)
Supplement: Supplementary file 1 [file Table1.docx]

| pathogens | Abbreviation | GenomeLabTM GeXP  ±1.5nt |
| --- | --- | --- |
| Influenza A viru | InfA | 105.0 |
| Influenza A virus H1N1 | InfAH1N1 | 163.3 |
| Seasonal Influenza virus H3N2 | H3 | 244.9 |
| Influenza B virus | InfB | 212.7 |
| Human Adenovirus | HADV | 110.2/113.9 |
| Boca virus | Boca | 121.6 |
| Human Rhinovirus | HRV | 129.6 |
| Human Parainfluenza virus | HPIV | 181.6 |
| Human Coronavirus | HCOV | 265.1 |
| Human Respiratory Syncytial virus | HRSV | 280.3 |
| Human Metapneumovirus | HMPV | 202.8 |
| Mycoplasma Pneumoniae | MP | 217.0 |
| Chlamydia | Ch | 190.5 |
| Internal Control | IC | 315.9 |

**Table S1 The details of Gexp analysis of 13 pathogens**
